# Cancer associated missense mutations in BAP1 catalytic domain induce amyloidogenic aggregation: A new insight in enzymatic inactivation

**DOI:** 10.1038/srep18462

**Published:** 2015-12-18

**Authors:** Sushmita Bhattacharya, Pranita Hanpude, Tushar Kanti Maiti

**Affiliations:** 1 Laboratory of Proteomics and Cellular Signaling, Regional Centre for Biotechnology, NCR Biotech Science Cluster, 3rd Milestone Gurgaon-Faridabad Expressway, Faridabad, Haryana 121001, INDIA; 2 Department of Biotechnology, Manipal University, Karnataka, 576104, INDIA

## Abstract

BRCA1 associated protein 1 (BAP1) is a nuclear deubiquitinase that regulates tumor suppressor activity and widely involves many cellular processes ranging from cell cycle regulation to gluconeogenesis. Impairment of enzymatic activity and nuclear localization induce abnormal cell proliferation. It is considered to be an important driver gene, which undergoes frequent mutations in several cancers. However the role of mutation and oncogenic gain of function of BAP1 are poorly understood. Here, we investigated cellular localization, enzymatic activity and structural changes for four missense mutants of the catalytic domain of BAP1, which are prevalent in different types of cancer. These mutations triggered cytoplasmic/perinuclear accumulation in BAP1 deficient cells, which has been observed in proteins that undergo aggregation in cellular condition. Amyloidogenic activity of mutant BAP1 was revealed from its reactivity towards anti oligomeric antibody in HEK293T cells. We have also noted structural destabilization in the catalytic domain mutants, which eventually produced beta amyloid structure as indicated in atomic force microscopy study. The cancer associated mutants up-regulate heat shock response and activates transcription of genes normally co-repressed by BAP1. Overall, our results unambiguously demonstrate that structural destabilization and subsequent aggregation abrogate its cellular mechanism leading to adverse outcome.

Since last three decades considerable progress occurred in genome sequencing field that reveals genomic landscape of cancer^1^. Advancement of genomic studies showed that there are more than hundred genes altered due to intragenic mutation. These mutations are essential for oncogenic progression. In a specific tumor type there are some driver genes that regulate core cellular processes like cell fate, cell survival and genome integrity. In recent years, BRCA1 associated protein 1 (BAP1), a nuclear deubiquitinating enzyme has emerged as an important tumor suppressor protein, undergoes frequent mutations in different types of tumor and appears as one of the driver genes in cancer types like uveal melanoma^1^, mesothelioma^2^, renal cell carcinoma^3^, cholangiocarcinoma^4^ and melanocytic tumors^5^.

Human BAP1 is deubiquitinating enzyme consisting of 729 amino acid whose N-terminal catalytic domain (1–240) shows homology to other ubiquitin C-terminal hydrolase (UCH) enzymes^6^. The C-terminal domain binds to N-terminal RING domain of BRCA1 and regulates BRCA1 mediated tumor suppressor function. BAP1 also acts as a transcriptional regulator wherein it co-activates/co-represses E2F mediated cell cycle genes through direct interaction with Host Cell Factor-1 (HCF1), a co-activator of E2F transcription factor^7^,^8^,^9^. It is well known that *calypso*, a *Drosophila* homolog of BAP1 exists in a complex with the polycomb group (PcG) protein ASXL1 and serves as a polycomb repressive deubiquitinase (PR-DUB)^9^,^10^. Reconstituted recombinant *Drosophila* and human PR-DUB complexes remove monoubiquitin from H2A^11^. BAP1 represses genes involved in DNA replication and repair by interacting with Foxk2 and inhibiting H2A monoubiquitination. It also plays a crucial role in double strand DNA break repair through homologous recombination (HR) and it is established that BAP1 acts as a DNA damage signaling and repair enzyme^12^,^13^. Mutations in catalytic domain fail to recruit BAP1 in the double strand DNA damage site and impair HR mediated repair^14^ leading to genomic instability, a hallmark in cancer pathogenesis^11^,^14^,^15^,^16^,^17^,^18^.

All types of mutation like insertions, deletions, frameshift, nonsense and missense occur in BAP1 gene located in chromosome 3p21.1, which is a hotspot region^5^,^19^,^20^,^21^,^22^,^23^,^24^,^25^,^26^,^27^. While BAP1 and its involvement in cancer is typically caused by mutations that lead to loss of protein function or deletion of key regulatory domains, missense mutations are more prevalent in tumors^1^,^25^. Catalogue Of Somatic Mutations In Cancer (COSMIC) database showed that out of 108 disease-associated mutations identified 69 (~60%) are located in the catalytic domain of BAP1. It is well known that hot spot mutations of any gene or predisposition of genetic elements has severe consequences on structure and function of protein^23^,^28^. Structural destabilization of proteins and its role in growing number of human diseases specifically neurodegeneration and cancer are well-established^29^,^30^,^31^,^32^,^33^. Uncontrolled overexpression or structural instability caused by mutations contributes to gain of toxic function and shows dominant negative effects^34^,^35^,^36^,^37^. There are evidences of missense mutations in cancer where a single change of an amino acid residue plays a major role in tumor progression^38^,^39^,^40^,^41^,^42^. The well known tumor suppressor protein p53 undergoes beta amyloid aggregation due to point mutations which has been studied thoroughly for the last few years. The molecular mechanism of aggregation of p53 in single molecule level has also been elucidated^38^,^41^,^43^,^44^. Destabilizing mutants of p53 not only aggregate themselves but also induce the aggregation of wild type protein^45^. It also coaggregates with other proteins like p63 and p73 in different types of cancer^46^. Recently, small molecules are identified to stabilize the aggregating mutants of p53 which might play a role in improvement of cancer^44^,^47^. However, it remains unexplored how protein aggregation caused by alteration of protein structure particularly oncogene or tumor suppressor leads to induction of malignancy.

To address these questions, we have screened missense mutations in genomic landscape of BAP1 and found that catalytic domain mutations play an important role in oncogenic progression. In the present report, we have demonstrated for the first time that cancer associated catalytic domain mutants (I47F, F81V, A95D and G178V) of BAP1 show a loss of enzymatic activity, significantly decreased protein stability and subsequently lead to beta amyloid aggregation *in vitro*. Up-regulation of heat shock response and changes in transcriptional activity of genes related to cell cycle control occur as a consequence of BAP1 inactivation. Our results indicate loss of BAP1 function due to aggregation.

## Results

**Figure 1:**
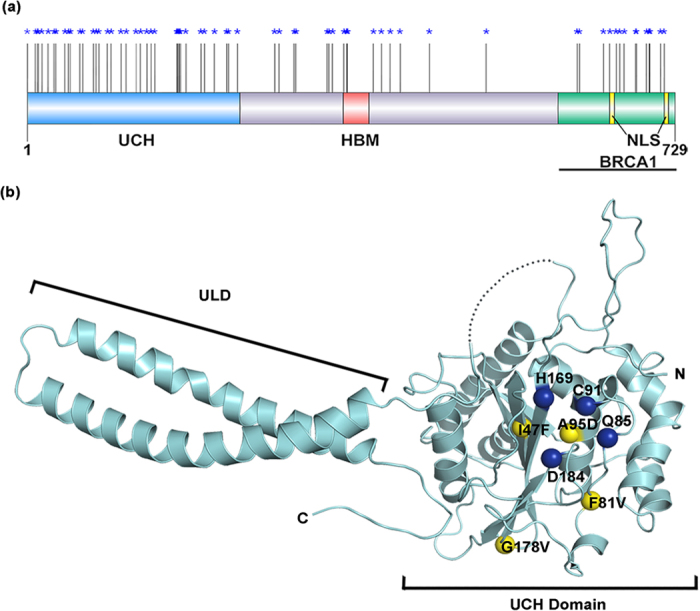
Missense mutations frequently occur in the catalytic domain of BAP1. (**a**) Location of point mutations in BAP1 was observed in a simplified structure of BAP1 according to COSMIC database. (**b**) Predicted BAP1 structure was generated by PyMOL based on UCHL5 crystal structure. Four important missense mutants (I47F, F81V, A95D and G178V) are denoted as yellow. Blue symbolizes important amino acid residues in the catalytic triad of BAP1.

Sequence alignment of all UCH members of deubiquitinases showed that there are number of amino acids conserved in the UCH domain from flies to human and also among the family members, indicating their critical role in protein structure and function (Supplementary Fig. S1a, b). Domain wise distribution of all missense mutants of BAP1 in cancer and location of the mutations in UCH domain are shown in Fig. 1a. Earlier studies reported that A95D and G178V mutations impaired catalytic activity in lung cancer^48^. However the molecular basis of inactivation has not been elucidated. Here, we have included two other cancer-associated mutants I47F and F81V, which are also found in different types of cancer^16^. The homology model structure of BAP1 predicts that A95D position is close to catalytic cysteine residue and in the same helix whereas I47F, F81V and G178V mutants are away from catalytic site (Fig. 1b). One may argue that the positioning of mutations in the rigid catalytic cleft disrupts the structural fold and that could be the possible reason for catalytic inactivation. However, there are no biochemical studies so far. Here we have attempted to explain the molecular basis of enzymatic inactivation of BAP1 upon mutation.

### Cytoplasmic sequestration of BAP1 in cancer associated mutants

**Figure 2:**
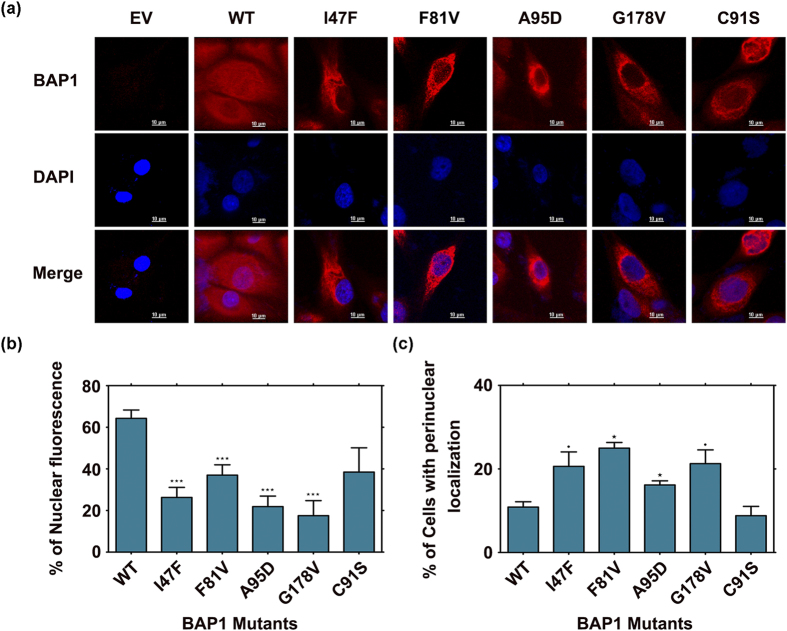
Differential cellular distribution of wild type BAP1 and its mutants. (**a**) Immunofluorescence microscopy was performed after 48 h of transfection in NCI-H226 lung cancer cells. Homogenous nuclear staining of BAP1 was observed in Flag-HA tagged wild type BAP1 (WT) transfected NCI-H226 cells. Mutants (I47F, F81V, A95D and G178V) revealed cytoplasmic accumulation/perinuclear localization of BAP1 protein. Empty vector (EV) transfected cells served as control. Scale Bars: 10 μm. (**b**) Statistical analysis for nuclear localization showed higher intensity of BAP1 in wild type. (**c**) Statistical analysis displayed perinuclear localization in mutants. Data represents mean values of three independent experiments °p < 0.1, ***p < 0.001, and *p < 0.05, WT.

To understand how a single amino acid change affects protein localization at the cellular level, site specific mutations have been incorporated in HA-FLAG-BAP1 plasmid by site directed mutagenesis. We transiently over expressed wild type and mutant BAP1 in human lung squamous carcinoma cell line NCI-H226, which is devoid of endogenous BAP1. Immunofluorescence study showed that cytoplasmic sequestration of BAP1 occurs due to point mutation in the catalytic domain (Fig. 2a). To our surprise catalytic dead mutant C91S showed less intensity of BAP1 in nucleus (Fig. 2b). It is interesting to note that the cancer-associated mutants I47F, F81V, A95D and G178V showed significantly lower nuclear localization than C91S mutant (Fig. 2b). On the other hand, these mutants showed much higher perinuclear accumulation as compared to C91S and wild type BAP1 (Fig. 2c). In our subsequent studies, we investigated BAP1 destabilization effect in cellular conditions as well as protein level.

### BAP1 mutants display prefibrillar aggregates in HEK293T cells

**Figure 3:**
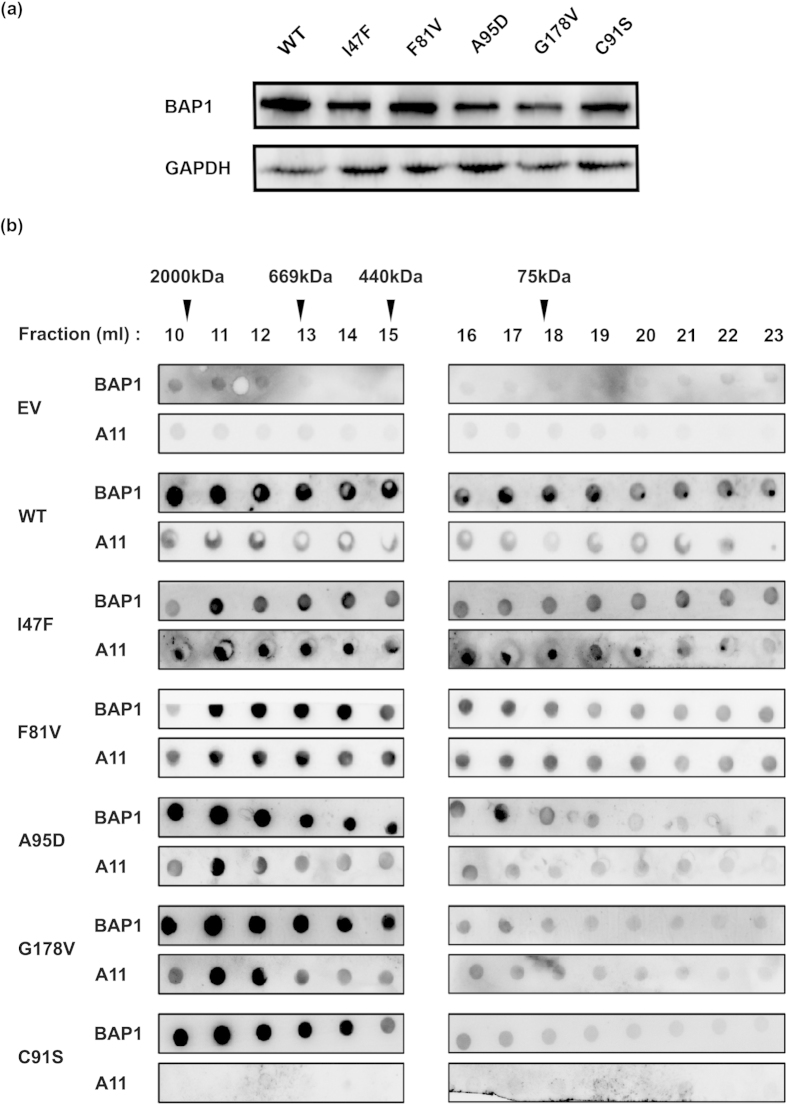
Characterization of BAP1 amyloid aggregates by dot blot assay. (**a**) HEK293T cells were transfected with Flag-HA wild type (WT), empty vector (EV) and mutant (I47F, F81V, A95D, G178V and C91S) plasmids. After 48 h of transfection, cells were harvested and immunoblot analysis was performed. GAPDH was used as a loading control. Similar expression level of wild type and mutant BAP1was detected. (**b**) Dot blot analysis of cellular extracts of BAP1 wild type (WT) and its mutants (I47F, F81V, A95D, G178V and C91S) was performed. Chromatographic fractions after gel filtration were dot blotted and incubated with anti-BAP1 and anti-A11 antibodies. Reactivity of A11 and BAP1 were normalized to total protein content. Molecular weight and fraction numbers are indicated in the upper panel.

Asymmetrical nuclear distribution of BAP1 prompted us to investigate the role of mutant BAP1 in perturbation of protein function. In earlier reports, there are predictions that the missense mutation induces protein structure destabilization^49^. Therefore, to examine the mechanism behind cytoplasmic accumulation of BAP1 we transiently overexpressed empty vector (EV), BAP1 wild type (WT), I47F, F81V, A95D, G178V and C91S mutants in HEK293T cells. All mutants showed similar expression level (Fig. 3a). Size exclusion chromatography was performed to fractionate cellular extracts of wild type and mutants. Each chromatographic fraction was subjected to dot blot analysis with anti-BAP1 antibody (Fig. 3b, Supplementary Fig. S2). Dot-blot analysis showed that wild type protein eluted in wide range of molecular weight fractions from 2,000 kDa to 70 kDa due to formation of multi-protein complex. On the other hand, A95D, G178V and C91S mutants were eluted from void volume to 400 kDa. To characterize the nature of high molecular weight fractions we have tested the reactivity of each chromatographic fraction with anti-A11 antibody, which specifically reacts with prefibrillar amyloid aggregates. Wild type and catalytically inactive mutant C91S showed less reactivity towards A11. Conversely, cancer associated mutants showed high reactivity with A11 (Fig. 3b). To verify the destabilization effect of these mutants we have centrifuged cellular extracts of wild type and mutants at 1,00,000 × *g*, separated pellet and supernatant fractions. Pellet was dissolved in equal amount of SDS sample buffer and analyzed by western blot. Wild type and C91S mutants were detected more in soluble fraction while I47F, F81V, A95D and G178V were observed more in pellet fraction (Supplementary Fig. S3). Together with microscopic data, these experiments further demonstrated aggregation of mutant BAP1 in cellular conditions.

### Loss of deubiquitinase activity of mutant BAP1 is associated with structural instability

**Figure 4:**
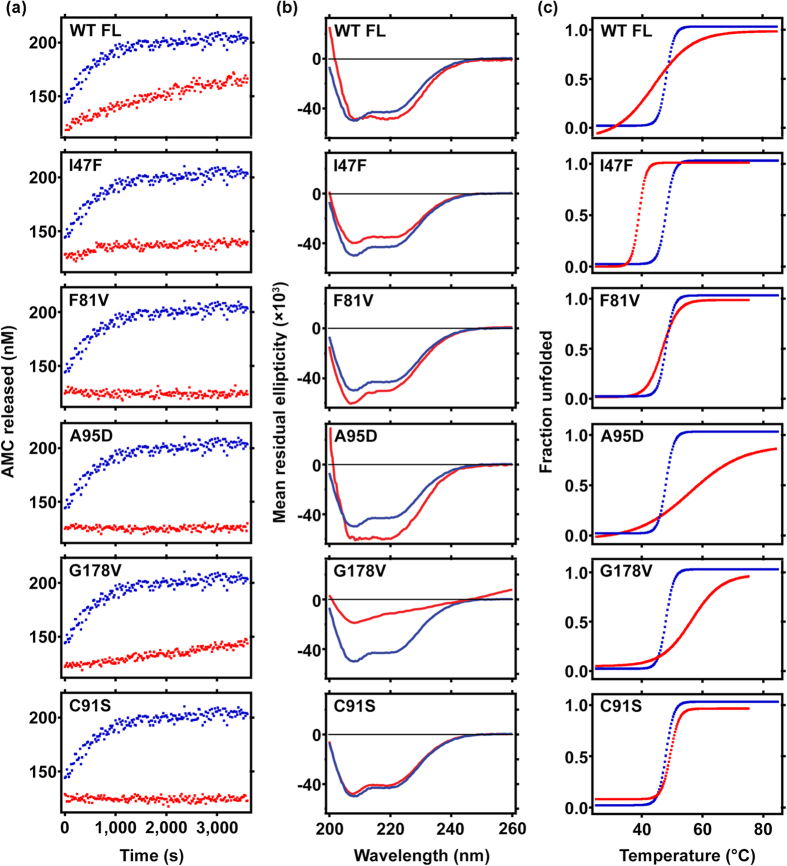
Comparative analysis of enzymatic activity, secondary structure and thermal stability of wild type BAP1 (1–240) with full length BAP1 and catalytic domain mutants. (**a**) Progress curve shows Ub-AMC hydrolysis by BAP1 wild type (WT) and all catalytic domain mutants (I47F, F81V, A95D, G178V and C91S). Substrate and enzyme concentrations were 600 nM and 250 pM for all reactions respectively. The amount of AMC released from the substrate was estimated using concentration dependent plot of AMC. (**b**) Circular dichroism spectra at 25 °C of full length wild type BAP1 (WT FL), catalytic domain wild type BAP1 (WT 240) and catalytic domain mutants showed characteristic secondary structure between 200–260 nm. (**c**) Complete temperature unfolding profile at 222 nm demonstrated distinct unfolding nature of full length BAP1 (WT FL) and mutants in comparison with wild type catalytic domain BAP1 (WT 240). Melting temperatures were obtained from sigmoidal fits over the range 24 °C–84 °C. Catalytic domain BAP1 (WT 240) is denoted as blue curve. All mutants and full length BAP1 (WT FL) are represented as red curve.

For better understanding of the physiochemical behavior of BAP1 mutants, His-tag BAP1 (1–729) was cloned, expressed and purified. Expression of full-length protein in *E. coli* was considerably low hence all biochemical and enzymatic experiments were carried out using the catalytic domain of BAP1 (1–240). Catalytic domain of BAP1 was subcloned into pGEX-6PI vector and protein was purified according to standard GST purification protocol. I47F, F81V and C91S mutants were purified similarly as wild type protein however A95D and G178V appeared mostly in pellet fraction after homogenization and centrifugation. Therefore, we modified the purification protocol by adding 0.1% TritonX100 and 5% glycerol during suspension. To examine *in vitro* effect of mutation on enzymatic activity of BAP1 we performed deubiquitinase assay using a model substrate, Ub-AMC. Wild type BAP1 (1–240) showed higher enzymatic activity as compared to full length BAP1 (WT FL). Lower enzymatic activity of BAP1 full length might be due to auto-inhibition by C-terminal domain (UCH L5 like domain or ULD domain). F81V and A95D showed a complete loss of enzymatic activity while I47F and G178V reflected basal level activity (Fig. 4a), which is probably due to perturbation of hydrophobic packing in the core structure. Inhibition of enzymatic activity by the mutants prompted us to further investigate mutational effect on protein structure.

We conducted circular dichroism (CD) spectroscopy to examine the effect of mutation on the secondary structure of BAP1 catalytic domain. Secondary structure analysis showed differences in curves of I47F and A95D. However, G178V mutant showed a complete destabilization effect (Fig. 4b). In order to understand the mutational effect on protein stability, we performed thermal melting analysis of wild type and mutant BAP1 (1–240). The cancer-associated mutants induce thermal instability, which is reflected in Tm values. A sharp transition curve was observed in wild type catalytic domain, C91S and F81V with a melting temperature of ~48 °C, 49 °C and 47 °C respectively (Fig. 4c). I47F mutant exhibited identical sharp transition curves with lowering of Tm value (~39 °C) (Table 1) Sharp transitions are associated with cooperative unfolding where initially the protein exists as a highly compact well-folded structure whereas a gradual non-cooperative transition indicates that the protein exists as a flexible, partially unfolded or heterogeneous population of folded structure. A95D, G178V and BAP1 full length do not show cooperative melting curve but they aggregate rapidly as temperature is increased. Circular dichroism analysis revealed increase in beta sheet after heating wild type and mutant proteins (Supplementary Fig. S4). Circular dichroism experiment results proved that the cancer-associated mutants destabilize the protein structure, which eventually translates into catalytic function loss.

### Analysis of aggregation propensity of BAP1

**Figure 5:**
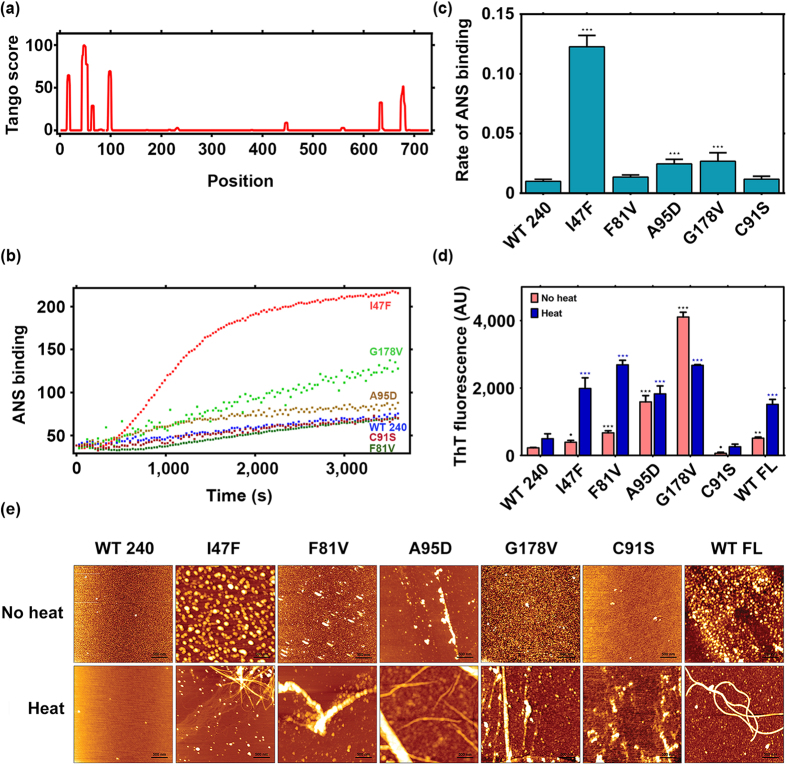
Characterization of fibrillar aggregates of BAP1. (**a**) Tango prediction analysis displays aggregation prone sequences of BAP1 from 13–20, 43–52, 78–100 amino acid residues in the catalytic domain and 656–680 residues in the C terminal region. (**b**) ANS fluorescence experiments display extent of exposure of hydrophobic patches in wild type and mutant BAP1. (**c**) Bar diagram represents initial rate of aggregation kinetics of BAP1 as monitored by ANS fluorescence. (**d**) ThT fluorescence was used to detect aggregation propensity of BAP1. 20 μM of control (no heat) and heat induced BAP1 wild type (WT 240), full length BAP1 (WT FL) and mutants (I47F, F81V, A95D, G178V and C91S) were incubated with 20 μM ThT for 15 min. ThT fluorescence emission intensity shows binding of BAP1 aggregates. (**e**) AFM images of BAP1 (WT 240), I47F, F81V, A95D, G178V, C91S and full length BAP1 (WT FL) displayed amyloid fibril formation after heat induction. BAP1 (WT 240) and its inactive mutant C91S displayed spherical aggregates whereas I47F, A95D, G178V, and BAP1 (1–729) showed fibrillar aggregates of height ranging from 2–10 nm. Scan size of the AFM image was 3.0 μm x 3.0 μm. Representative results of three independent experiments. °p < 0.1, ***p < 0.001, **p < 0.01 and *p < 0.05.

To understand why structurally destabilizing mutations in BAP1 induce oligomerization, we used TANGO, an algorithm for predicting protein aggregation sequences and to identify regions in protein that has more oligomerization propensity. Here, we have identified a few prone segments that span residues from 13–20, 43–52, 78–100 and 656–680. These residues are mostly hydrophobic. The segment comprising residues from 43–52 shows the highest TANGO score representing maximum tendency to form beta aggregates (Fig. 5a). The homology model structure of BAP1 catalytic domain shows that aggregation prone regions lie in hydrophobic core of protein. Mutation in catalytic domain of BAP1 destabilizes its secondary structure, is likely to expose the regions that are normally buried in hydrophobic core, such as the aggregation-nucleating region. So these mutations are also prone to trigger aggregation of BAP1 by assembly of beta amyloid type aggregates into a sheet like structure.

### BAP1 mutants showed beta amyloid aggregation *in vitro*

Based on the melting behavior of wild type and mutants; we determined misfolding characteristics of BAP1 protein. Exposed hydrophobic surface of a protein plays a significant role in protein aggregation and change of single amino acid residue may alter the physico-chemical property of specific site leading to structural destabilization. Therefore, we have investigated the differential binding behaviors of wild type and mutant proteins with ANS dye. ANS specifically binds to exposed hydrophobic surfaces which is necessary for the initiation of oligomer formation^50^. ANS binding results revealed a rapid exposure of hydrophobic surfaces in mutants of BAP1 as temperature is increased. The I47F mutant showed highest change of fluorescence intensity starting with a lag phase indicating association of monomers with oligomers and then rapid increase in the log phase, which indicates acceleration of aggregation kinetics (Fig. 5b,c). F81V, A95D and G178V showed increase in ANS binding compared to wild type BAP1 while C91S behaves like BAP1 wild type protein. We have also measured binding of BAP1 protein to Thioflavin-T (ThT) dye, which binds specifically to ordered aggregates. Comparison of ThT binding activity with BAP1 wild type and mutants was examined at 25 °C and heat induced condition (37 °C). Enhancement of ThT fluorescence of mutant protein after heating indicates ordered aggregate formation. It is interesting to note that full length BAP1 also binds with ThT confirming its inherent amyloidogenic character (Fig. 5d).

We have characterized morphology of protein aggregates after heat-induced condition by atomic force microscopy. All cancer associated mutants showed fibrillar aggregates upon heating though the extent of fibrillization varies in each mutant (Fig. 5e). Full length BAP1 (1–729) also exhibited fine fibrillar structure when heated. It is evident from TANGO analysis that BAP1 has several beta propensity regions mostly in catalytic and ULD domain. Consensus structure prediction also revealed that the middle region of BAP1 is mostly unstructured. This might be the probable reason for heat induced aggregation behavior of full length BAP1. In fact, the fibrillar structure of full length BAP1 corroborates with Tm analysis as indicated in Fig. 4c. In summary, BAP1 resembles amyloidogenic properties of aggregation prone proteins and instigated us to investigate loss of functional activity of BAP1 due to mutation.

### Cellular toxicity of mutant BAP1

**Figure 6:**
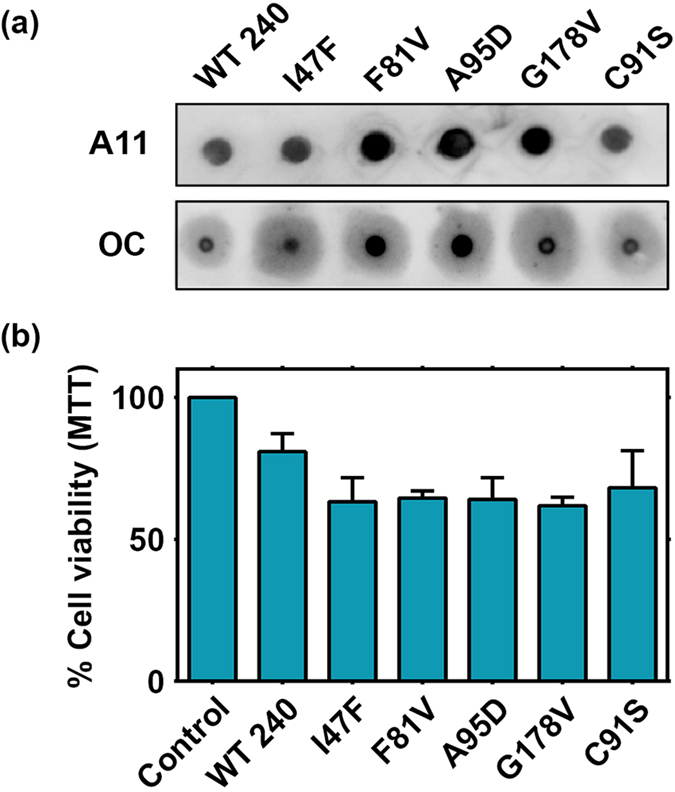
Cytotoxicity of BAP1 aggregates. (**a**) Heated wild type (7 μM) and mutant BAP1 solution was ultracentrifuged and the pellet containing aggregates of BAP1 was subjected to dot blot assay by anti-A11 and anti-OC antibodies. (**b**) Aggregates of wild type and mutant BAP1were added to HEK293T cells and cell viability was measured by MTT reduction assay after 24 h. Data represents results of four independent experiments.

Oligomer A11 antibody specifically binds to the prefibrillar protein aggregates and here we have demonstrated that BAP1 and cancer associated mutants overexpressed in HEK293T cells showed strong reactivity against A11. It has been established that cells exposed to prefibrillar aggregates or beta amyloid aggregates induce cell death response. Here we have tested the effect of BAP1 catalytic domain mutant aggregates on cellular toxicity. Prefibrillar aggregates of BAP1 (1–240) wild type and mutants were generated by heat induction at 37 °C. All mutants showed higher reactivity against A11 compared to BAP1 (1–240) wild type and its C91S mutant (Fig. 6a). We also tested the reactivity of the same aggregates with OC antibody, which specifically binds to amyloid fibrils. BAP1 wild type and mutant aggregates also showed reactivity against OC. Our results demonstrated that in the experimental condition there is a mixed population of prefibrillar and fibrillar aggregate which corroborates with our AFM results. Cellular toxicity of prefibrillar aggregates was tested by cell viability assay in HEK293T cells. Cells exposed to mutant aggregates showed higher mortality (Fig. 6b) in MTT assay.

### Aggregation of mutant BAP1 up-regulates heat shock protein response

**Figure 7:**
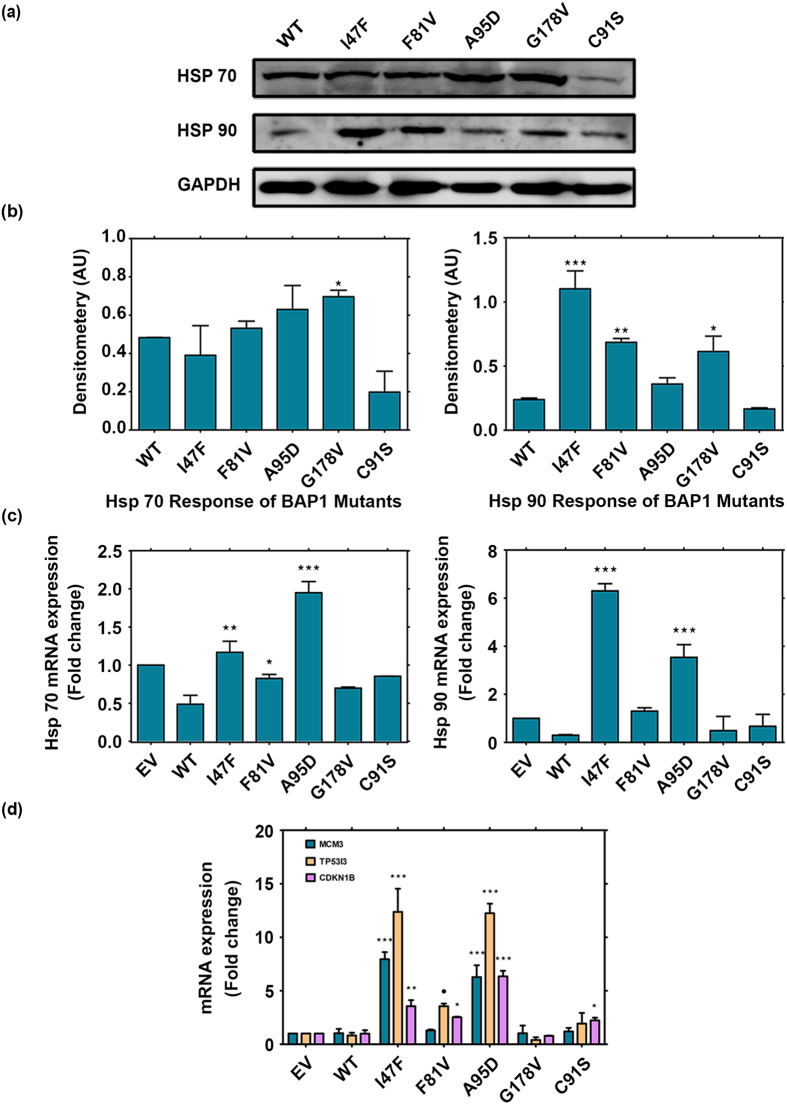
Overexpression of mutant BAP1 upregulates Hsp response and increases transactivity of BAP1. (**a**) Immunoblot analysis demonstrated increase of Hsp90 and Hsp70 at cellular level due to aggregation. GAPDH was used as a loading control. (**b**) I47F, F81V, G178V induced significantly higher level of Hsp90 as compared to wild type (WT). Substantial increase of Hsp70 was observed in aggregating mutants A95D and G178V. (**c**) Real time PCR analysis determined up-regulation of Hsp70 and Hsp90 at gene level. Values were normalized to GAPDH. Both I47F and A95D showed higher level of Hsp90 gene expression. A95D displayed substantial increase in Hsp70. Representative results are of four independent experiments. EV represents empty vector transfected cells. ***p < 0.001, **p < 0.01 and *p < 0.05, WT. (**d**) Q PCR analysis showed transactivation of *MCM3, TP53I3* and *CDKN1B* gene expression in mutants I47F, F81V and A95D as compared to wild type (WT) in HEK293T transfected cells. Wild type BAP1 normally acts as a repressor for these genes. Values were normalized to *GAPDH*. Representative results of three independent experiments °p < 0.1, ***p < 0.001, **p < 0.01 and *p < 0.05, WT.

The question remains however elusive as how aggregation of mutant BAP1 is linked to cancer pathogenesis. The heat shock proteins specifically Hsp70 and Hsp90 are known to be overexpressed in cancers and several chaperone proteins promote tumor cell proliferation while inhibiting cell death pathway^34^,^51^. Hsp90 acts directly upon amyloidogenic substrates^52^. Hsp70 is found to be associated with misfolded proteins and overexpressed in metastatic cancer^53^. Chaperones are critical to guide protein folding and their up-regulation enhances oncogenic progression. They help in modulating destabilized structure and facilitate inhibition of oligomer formation. Protein aggregation is one of the strong trigger factors of heat shock response and BAP1 might acquire the tumorigenic properties through the activation of heat shock proteins^54^. The cancer-associated mutants and BAP1 wild type were overexpressed in HEK 293T cells to assess Hsp90 and Hsp70 expression by western blot. I47F and F81V showed almost 1.5 to 3.0 fold up-regulation in Hsp90 protein levels with a marginal up-regulation in Hsp70 (Fig. 7a,b). qPCR analysis of Hsp70 and Hsp90 also corroborated with western blot analysis (Fig. 7c). Molecular chaperones are known to be up-regulated transcriptionally during misfolding response^55^. Therefore, up-regulation of Hsps at gene level in BAP1 mutation may drive tumorigenesis.

### Impact of missense mutations on transcriptional regulation

It is well known that BAP1 is associated with chromatin and acts as a co-activator/co-repressor for its target genes. It is recognized as an important regulator protein that dynamically controls transcriptional responses. It controls activation and deactivation of genes related to DNA replication and repair by interacting with transcription factors like Foxk2^56^. To determine the effect of mutants on transcriptional activity, we have transfected HEK293T cells with wild type and mutant plasmids. We performed quantitative PCR on genes which are regulated by BAP1 (*MCM3, CDKN1B* and *TP53I3*)^11^,^56^. MCM3 is a protein required during initiation of DNA replication, *CDKN1B* is a cell cycle regulator gene and *TP53I3* is up-regulated during cellular stress. Therefore, transactivity of these genes are important in regulation of oncogenesis. We found that mutants I47F, F81V and A95D significantly increased the expression of genes where BAP1 acts as a co-repressor (Fig. 7d). I47F and A95D augmented expression of *MCM3, CDKN1B* and *TP53I3* to three fold as compared to wild type. F81V and catalytic inactive mutant C91S showed marginal increase in expression of these genes. To our surprise, G178V did not show any impact on gene regulation. Collectively, these results suggest mutational loss of BAP1 can lead to dominant negative effect. Role of BAP1 in gene silencing has important implications in its anti-tumorigenic potential. Therefore, loss of BAP1 function due to mutation might lead to cancer progression.

## Discussion

Previous reports of BAP1 mutations showed wide range of consequences on tumor suppressor activity that contributes to oncogenic progression^4^,^5^. The effects of these mutations are highly diverse including loss of deubiquitinase activity, aberrant nuclear trafficking to cell cycle progression^8^,^16^,^48^. Different types of mutations are found but missense type is predominant in UCH domain which is the core activity domain of BAP1^1^,^57^. Here, we report that BAP1 protein undergoes aggregation due to mutation at protein and cellular level. Oligomerization propensity of BAP1 in catalytic domain is due to presence of abundant hydrophobic patches. Minor alterations in these hydrophobic regions expose a stretch of aggregation prone sequence leading to structural destabilization as observed in the ANS binding study. In BAP1 null cells, mutant protein accumulates in cytoplasmic region and impairs nuclear localization. The question arises what disturbs nuclear localization of BAP1. The mono ubiquitination of BAP1 by UBE2O ligase induces its cytoplasmic stay and auto deubiquitination of BAP1 protects it from cytoplasmic sequestration^58^. Our observations revealed that auto deubiquitination activity is not only responsible for contributing to cytoplasmic sequestration or perinuclear accumulation. Cytoplasmic sequestration or perinuclear localization as a consequence of protein aggregation is a well-known phenomenon and it is observed in several proteins like p53, Htt proteins, ataxin-3 and TDP43^34^. It is known that misfolded proteins undergo oligomerization to form high molecular weight aggregates that perturb nuclear trafficking of proteins. Thus BAP1 aggregation could be one of the causes for cytoplasmic sequestration or perinuclear accumulation for cancer associated mutants. In cellular condition, reactivity of oligomer specific antibody (A11) with cancer-associated mutants of BAP1 demonstrated that they form prefibrillar aggregates. These prefibrillar aggregates induce cellular toxicity, which is a characteristic behavior for aggregation prone protein.

Our biochemical study revealed that loss of enzymatic activity of BAP1 is associated with mutation in the catalytic domain. The thermal melting and circular dichroism studies have indicated that cancer associated mutations destabilize protein structure leading to the loss of activity. Circular dichroism analysis showed that there is an increase in beta sheet or disordered structure in mutants as compared to wild type when heated. In heat induced condition, catalytic domain protein aggregates also showed binding with A11 antibody confirming its prefibrillar nature which eventually produce more rigid beta amyloid aggregates as evident from OC antibody reactivity, ThT binding and AFM studies. These prefibrillar aggregates of BAP1 induce cytoxicity in HEK 293T cells which is contrary to its tumor suppressor function. But aggregation of p53 also induces cell death in cells. Gain of toxic function is important incase of amyloid diseases and cancer is now considered to be one of the amyloid specific diseases^59^. p53 induced cell death releases toxic aggregates that may cause chemoresistance in some kind of tumors or they may be transmitted for immortalization^60^.

Our results also lend support to the consequences of aggregation in functional activity of BAP1. We found that mutant BAP1 up-regulated heat shock response. Activation of Hsp70 and Hsp90 both at protein and gene level was observed. Heat shock proteins act as biochemical buffer to maintain stability of cancer cells^52^. Therefore, activation of this response due to conformational change in BAP1 links mutational effect of BAP1 with cancer. Another important aspect of BAP1 mutation towards functional loss is impairment of regulatory role of BAP1 in gene transcription^6^,^11^,^56^. Here, mutants of BAP1 showed higher levels of *MCM3, CDKN1B* and *TP53I3* expression where BAP1 acts as a co-repressor. BAP1 plays a major role in gene silencing mechanism by deubiquitinating H2A along with other interacting partners like Foxk2. Here, missense mutations in BAP1 increase transcriptional activity. Transcription of specific genes like *MCM3* and *CDKN1B* effects tumor suppressor ability of BAP1. TP53I3 is a regulator of cellular stress and activation of this gene due to BAP1 inactivation might aggravate oncogenic activity. So, these results highlight negative effect of BAP1 mutations at cellular level.

**Figure 8:**
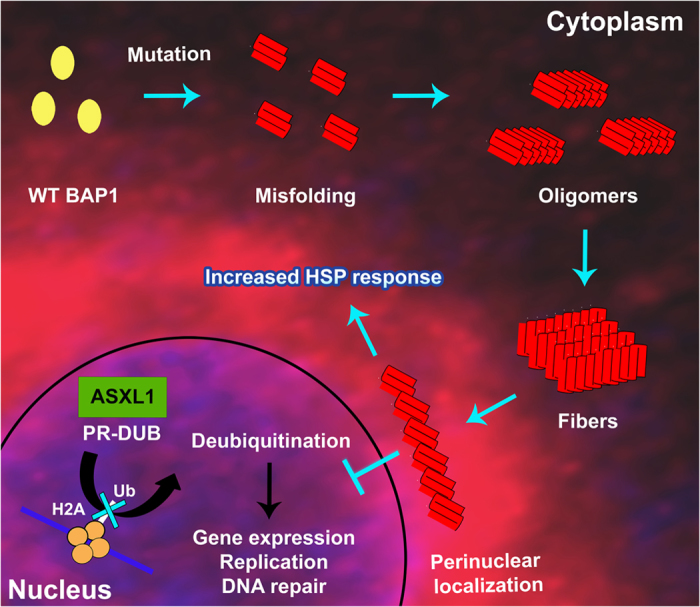
Schematic model representing BAP1 mutation and its effect in functional loss. Native and mutant conformations of BAP1 are represented as yellow and red respectively. Mutant BAP1 forms stable oligomers to induce fibril formation. Aggregation of BAP1 inhibits nuclear localization of BAP1, which prevents the PR-DUB complex formation and subsequent histone H2A deubiquitination.

Our study unambiguously demonstrated that cancer associated catalytic domain BAP1 mutations destabilize protein structure leading to formation of beta amyloid aggregates. Aggregation of protein and its association with cancer biology are not very well understood. However, there are a few examples where tumor suppressor protein p53 showed beta aggregation properties^34^. In fact, prion and protein-only inheritance in cancer is recently appreciated^32^. Amyloidogenicity of BAP1 will inevitably strengthen the prion hypothesis in cancer biology; however a detailed molecular study is necessary to understand its aggregation mechanism. Here we have hypothesized a schematic model showing that structural destabilization of BAP1 after mutation in the catalytic domain leads to aggregation followed by cytoplasmic sequestration and subsequent functional loss (Fig. 8). According to our hypothesis, oligomerization of mutant BAP1 inhibits PR-DUB complex formation and deubiquitination of histone 2A due to loss of its catalytic activity and impairment of nuclear transport that culminates into enhancement of oncogenic activity thereby explaining the effect of missense mutations in BAP1. Finally, correlating BAP1 mutation and cancer outcome at molecular level will help us to understand tumor biology and potential therapeutic development to combat cancer in general.

## Methods

### Cloning, expression and purification of BAP1

Flag-HA tagged full length BAP1 was obtained from Addgene, USA. Catalytic domain of BAP1 (1–240) and full length BAP1 (1–729) were sub-cloned into pGEX-6P-1 and pET28a vector respectively using standard cloning procedure. Mutations on the catalytic domain of BAP1 and full length Flag-HA BAP1 were generated by site directed mutagenesis using Quick-change site directed mutagenesis kit (Stratagene, Santa Clara, CA, USA). Catalytic domain BAP1 (1–240) and all cancer-associated mutants were expressed in *E. coli* Rosetta 2 cells (Novagen, Gibbs Town, NJ, USA) and proteins were purified according to the standard GST purification protocol. Full length BAP1 was purified according to His-tag purification protocol. Briefly, BL21 (DE3) cells containing pET28a-BAP1 plasmid DNA were grown to 0.8 O.D and then induced with 20 μM/L of IPTG at 15 °C for 24 h. Cells were harvested and pellet was resuspended in lysis buffer containing 50 mM Tris-HCl, pH 8.0, 1 mM MgCl_2_ and 10 μg/ml lysozyme. Homogenized solution was loaded on to a TALON Metal Affinity Resin (Clonetech) that was previously equilibrated with 50 mM Tris-HCl, pH 8.0, 150 mM NaCl, 10 mM imidazole. Protein was eluted in 50 mM Tris-HCl, pH 8.0, 300 mM NaCl, 200 mM imidazole. Eluted protein was dialyzed extensively with 50 mM Tris-HCl pH 8.0, 50 mM NaCl. Protein was concentrated and loaded on to Mono Q5/50 column. Eluted fraction was concentrated and verified by SDS-PAGE.

### Cell culture and transfection

Human lung cancer cell line NCI-H226 was obtained from ATCC and cultured in RPMI-1640 supplemented with 10% FBS, penicillin and streptomycin. Proliferating cells were maintained in a 5% CO_2_ incubator. HEK293T cells were maintained in DMEM supplemented with 10% FBS, penicillin and streptomycin. Transient transfection of plasmids was performed using lipofectamine 2000 following standard transfection protocol. Briefly, HEK293T cells were seeded in 150 cm^2^ flasks. DNA-lipofectamine complex was prepared in 1.75 ml of Opti-MEM media by mixing 60 μg of plasmid DNA with 120 μl of lipofectamine for 30 min before adding to 90% confluent cells. Cells were harvested after 48 h of transfection and dissolved in respective buffers for gel filtration, dot blot and western blot analysis. Immunofluorescence was performed by seeding NCI-H226 cells (1 × 10^6^) in Labtek chamber slides with subsequent transfection using 2 μg of plasmid DNA in 85–90% confluent cells. After 48 h of transfection, cells were examined and processed for immunofluorescence.

### Gel filtration, dot blot and immunoblot analysis

Oligomeric behavior of BAP1 in cellular condition was analyzed by size exclusion chromatography. Wild type and mutant BAP1 were overexpressed in HEK293T cells. Transfected cells were pellet down and dissolved in a buffer containing 50 mM Tris-HCl pH 7.4, 150 mM NaCl, 0.5% sodium deoxycholate and protease inhibitor mixture (Sigma Aldrich). Cell lysates were centrifuged for 5 min at 3,000 RPM and 350–400 μg of supernatant protein was immediately loaded onto a Superose 6 (10/300 GL) (GE Healthcare) gel filtration column, which was previously equilibrated in 50 mM Tris-HCl, 150 mM NaCl, 5 mM DTT, 5% glycerol buffer. Each chromatographic fraction was dotted on a nitrocellulose membrane using Bio Rad Bio Dot apparatus. Membranes were blocked in 3% skimmed milk for 1 h. After blocking membranes were incubated with anti-BAP1 antibody (1:1,000) (Santa Cruz Biotechnology, Inc., USA) and anti-A11 antibody (1:2,000) (Life Technologies, USA) for overnight. Subsequently, membranes were further incubated with anti-rabbit secondary antibody (conjugated to HRP) (Jackson Immuno Research Laboratories, Inc. Baltimore USA) for 2 h. Membranes were developed using Luminata Forte ECL reagent (Millipore, USA) and visualized in chemiluminescence system. Immunoblot analysis was performed in cellular extracts from transfected cells. Briefly, cells were lysed, denatured by 2% SDS sample buffer and loaded in 10% SDS-PAGE gel. After electrophoresis, gels were transferred to PVDF membranes. Membranes were incubated in 5% skimmed milk for 1 h and further incubated with primary antibodies for overnight. Antibodies anti-BAP1 (1:1,000), anti-GAPDH antibody (1:2,000) (Santa Cruz Biotechnology, USA), anti-Hsp70 (1:1,000) and anti-Hsp90 (1:1,000) (Thermo Scientific) were diluted in blocking buffer. Finally, membranes were incubated with respective secondary antibodies for 1 h and developed using Luminata Forte reagent. Immunoreactive bands were visualized by western blot detection system. (Image Quanta LAS 4000, GE). Densitometry analysis was performed using Quantity one 1-D analysis software (Bio-Rad Laboratories, USA).

### Immunocytochemistry

Transfected NCI-H226 cells were fixed with 4% paraformaldehyde. Fixed cells were rinsed with PBS and permeabilized with PBST (PBS, 1% BSA, 0.5% Triton X-100) for 30 min. Primary (anti-BAP1) antibody was diluted in blocking buffer (1:200) and incubated for overnight. Cells were further incubated with anti-rabbit secondary Cy3 antibody (1: 1,000) (Jackson Immuno Research Laboratories, Inc. Baltimore USA) for 1 h. After washing with blocking buffer, DAPI (1:10,000) staining was done and mounted with anti-fading reagent Prolong-Gold (Invitrogen, Life Technologies, USA). Images were acquired by 63x objective of confocal fluorescence microscope (Leica TCS SP5 II). Protein localization was quantified in wild type and mutants by analyzing multiple fields from three independent distinct experiments.

### Solubility assay

To quantify protein solubility in HEK293T cells, transient transfection was performed with wild type or mutant Flag-HA-BAP1. Cells were lysed in buffer containing 50 mM Tris-HCl pH 7.4, 150 mM NaCl, 0.5% sodium deoxycholate, protease inhibitor and total protein concentration was estimated. Lysed cells were centrifuged at 1,00,000 × *g* for 90 min. Supernatant and pellet fractions were subjected to western blot. Protein level was compared with the protein content in the supernatant and pellet fraction to the total fraction. Solubility was quantified using densitometry analysis software Quantity one 1-D analysis.

### Enzymatic activity assay

Stock solution of full length BAP1, BAP1 catalytic domain (1–240) and catalytic domain mutants I47F, F81V, C91S, A95D and G178V were diluted with reaction buffer containing 50 mM Tris-HCl, pH 7.6, 5 mM DTT, 1 mM EDTA, 0.1 mg/ml of BSA in the individual wells of 96-well plate to a final concentration of 250 pM. Ub-AMC (Boston Biochem Inc. Cambridge MA, USA) was added to each well at final concentration of 600 nM. Final reaction volume in each well was 100 μl. The rate of Ub-AMC hydrolysis was monitored at 30 °C for 60 min using SpectramaxM5 plate reader (Molecular Devices, USA). The excitation and emission wavelength used in this measurement were 355 nm and 455 nm respectively. The amount of AMC released due to the hydrolysis by BAP1 was quantified using 7-amido-4-methylcoumarin as a standard (Sigma Aldrich).

### Circular Dichroism Spectra analysis

Circular dichroism spectra for wild type and different mutants were recorded from 200 nm to 260 nm. Briefly, wild type and mutant proteins were dissolved in 50 mM Tris-HCl pH 7.4 to a final concentration of 5 μM, and the spectra were recorded using JASCO J815 CD spectrometer at different temperatures. Tm for each protein was estimated from the unfolded protein fraction at 222 nm for varying temperature.

### Atomic force microscopy

Full length BAP1, catalytic domain BAP1 (1–240) and catalytic domain mutants protein samples were diluted with 50 mM Tris-HCl pH 7.4, 50 mM NaCl to a final concentration of 10 μM. Protein sample (10 μM) was heat induced at 37 °C for 1 h followed by 14 h incubation at 25 °C. After induction, proteins were deposited on freshly cleaved mica and then dried for 30 min. Samples were imaged in tapping mode by JPK Nano Wizard III atomic force microscope. The drive frequency of silicon cantilever was between 290 to 320 kHz and the scan rate was between 0.5 to 1 Hz with a spring constant of 13–77 N/m. The length of fibrillar aggregates was measured from the topographic AFM images with JPK software.

### Thioflavin T (ThT) binding assay

ThT fluorescence was recorded using F-7000 fluorescence spectrophotometer (Hitachi High-Technologies Corporation) with fluorescence excitation at 412 nm and emission at 485 nm. Protein solution was dissolved in 50 mM Tris-HCl pH 7.4, 150 mM NaCl buffer incubated for 2 h at 37 °C heat followed by 2 h at 25 °C. Readings were taken from samples after incubating 20 μM of induced and control protein solution with 20 μM of ThT for 15 min. Three independent measurements were performed and subsequently averaged for each sample.

### ANS binding

ANS (8 anilino-1-naphthalene sulfonic acid) (20 μM) was mixed with 10 μM of protein in 50 mM Tris-HCl, 150 mM NaCl, pH 7.4 at 37 °C and increase of ANS fluorescence was monitored over 1 h with 30 s intervals using a SpectramaxM5 plate reader (Molecular Devices, USA). The excitation and emission wavelength were 350 nm and 460 nm respectively.

### Cytotoxicity assay

Wild type and mutant BAP1 (1–240) (400 μl of 7 μM) were heated at 37 °C for 1 h followed by 14 h incubation at 25 °C and protein aggregates were harvested using ultracentrifugation at 1,00,000 × *g* for 60 min. For OC reactivity, wild type and mutants were centrifuged for 2 h at 1,00,000 × *g.* Aggregates were dissolved in 200 μl of water and 15 μl of dissolved aggregates were dot blotted in a nitrocellulose membrane using BioRad dot blot apparatus. The membrane was incubated with A11 and OC antibodies for overnight at 1:1,000 dilution and subsequently incubated with HRP conjugated anti-rabbit secondary antibody (1:5,000) for 2 h. The membrane was washed with PBST and developed with Luminata Forte western HRP substrate (Millipore).

HEK293T cells (5 × 10^4^) were plated in 96 well plates. Cells were maintained in DMEM supplemented with 10% FBS and antibiotic solution at 37 °C in a 5% CO_2_ incubator. Protein aggregates were added at 1 μM concentration. After 24 h, MTT was added in the medium and kept at 37 °C for 4 h. The amount of reduced MTT (formazan) was solubilized in DMSO and cell survivability was determined by monitoring the absorbance at 562 nm in SpectromaxM5 Plate reader.

### Quantitative Reverse Transcription-PCR (RT-qPCR)

Effect of BAP1 mutation on up-regulation of Hsp70, Hsp90 and transactivation of three target genes (*MCM3, TP53I3* and *CDKN1B*) at cellular level was determined by RT-qPCR. HEK293T cells were transfected with empty vector (EV), wild type and mutant plasmids. After 48 h, total RNA was isolated using RNeasy mini kit (QIAGEN). Reverse transcription was performed using Verso cDNA Synthesis Kit (Thermo Scientific) according to the manufacturer’s protocol. Quantitative real-time PCR was determined by using Dynamo Flash SYBR Green qPCR kit (Thermo Scientific) on a C1000 Touch Thermal cycler real-time machine (Bio-Rad) (Supplementary Table 1).

## Additional Information

**How to cite this article**: Bhattacharya, S. *et al.* Cancer associated missense mutations in BAP1 catalytic domain induce amyloidogenic aggregation: A new insight in enzymatic inactivation. *Sci. Rep.* **5**, 18462; doi: 10.1038/srep18462 (2015).

## Supplementary Material

Supplementary Information

## Figures and Tables

**Table 1:** Melting point analysis of wild type and mutant BAP1 proteins.

S. No.	Name of Protein	Tm (°C)
1	WT 240	48.0 ± 0.1
2	WT FL	44.0 ± 0.6
3	I47F	39.1 ± 0.3
4	F81V	46.8 ± 0.2
5	A95D	55.9 ± 1.6
6	G178V	56.4 ± 0.9
7	C91S	49.5 ± 0.4
