# Supplementary material for: Cancer associated missense mutations in BAP1 catalytic domain induce amyloidogenic aggregation: A new insight in enzymatic inactivation
Source: Sci Rep. 2015 Dec 18;5:18462. doi: 10.1038/srep18462 (PMC4683529; doi:10.1038/srep18462)
Supplement: Supplementary Information [file srep18462-s1.doc]

**Supplementary Figure legends**

**Supplementary Figure S1.** **Catalytic domain of BAP1 is conserved.**  (a) BAP1 catalytic domain is conserved with other family members. Regions from 1-230 amino acids demonstrate high sequence similarity with other UCH members UCHL1, UCHL3 and UCHL5. (b) Sequence alignment of BAP1 depicts that catalytic domain of human BAP1is highly conserved with other related species like Drosophila (DROME), mouse (MOUSE), zebra fish (DANRE), chicken (CHICK), rat (RAT) and bovine (BOVIN). Amino acids 1-255 show alignment with different species. Regions from 356 to 393 amino acids display similarity but C terminal domain shows no alignment.

**Supplementary Figure S2.** **Gel filtration chromatogram profile of wild type and mutant BAP1.** Size exclusion chromatographic profile of HEK293T cellular extracts where BAP1 wild type and mutants are overexpressed. Blue curve represents wild type chromatogram and red curve represents mutant

**Supplementary Figure S3. Solubility of cellular BAP1**. (a) Cellular extracts from wild type (WT) and mutant BAP1 (I47F, F81V, A95D, G178V and C91S) transfected cells were centrifuged and subsequently pellet, soluble and total fractions were immunoblotted with anti-BAP1 antibody. (b) Compared to wild type significantly more BAP1 was in the pellet fraction of mutants. Representative results of three independent experiments. **p<0.01, *p<0.05 WT.

**Supplementary Figure S4.** **Secondary structure analysis of BAP1.** Bar diagram represents amount of α helix, β strand and disordered conformation present in heated and non-heated samples of WT and mutant BAP1.

**Supplementary Figure S1**


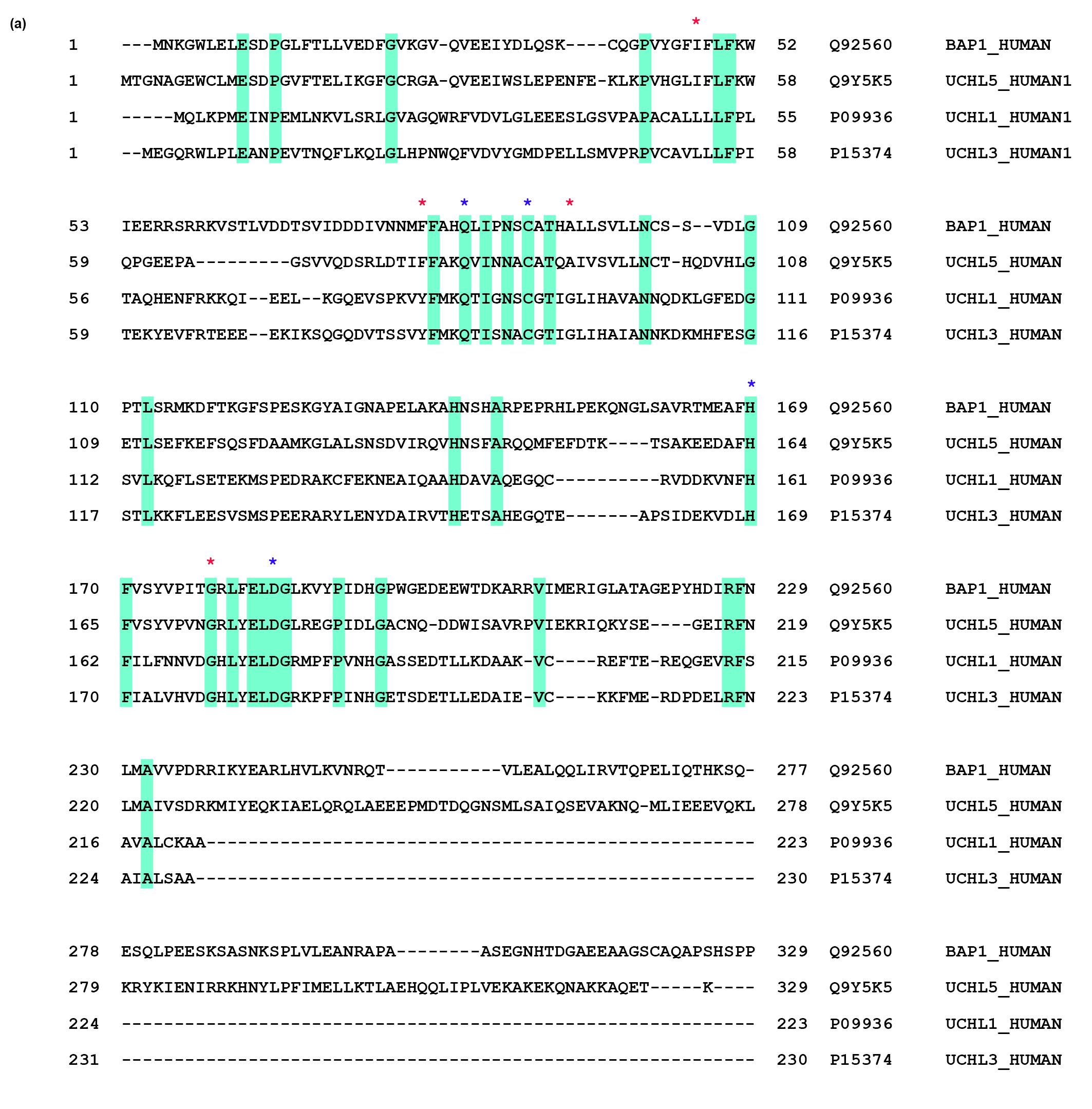


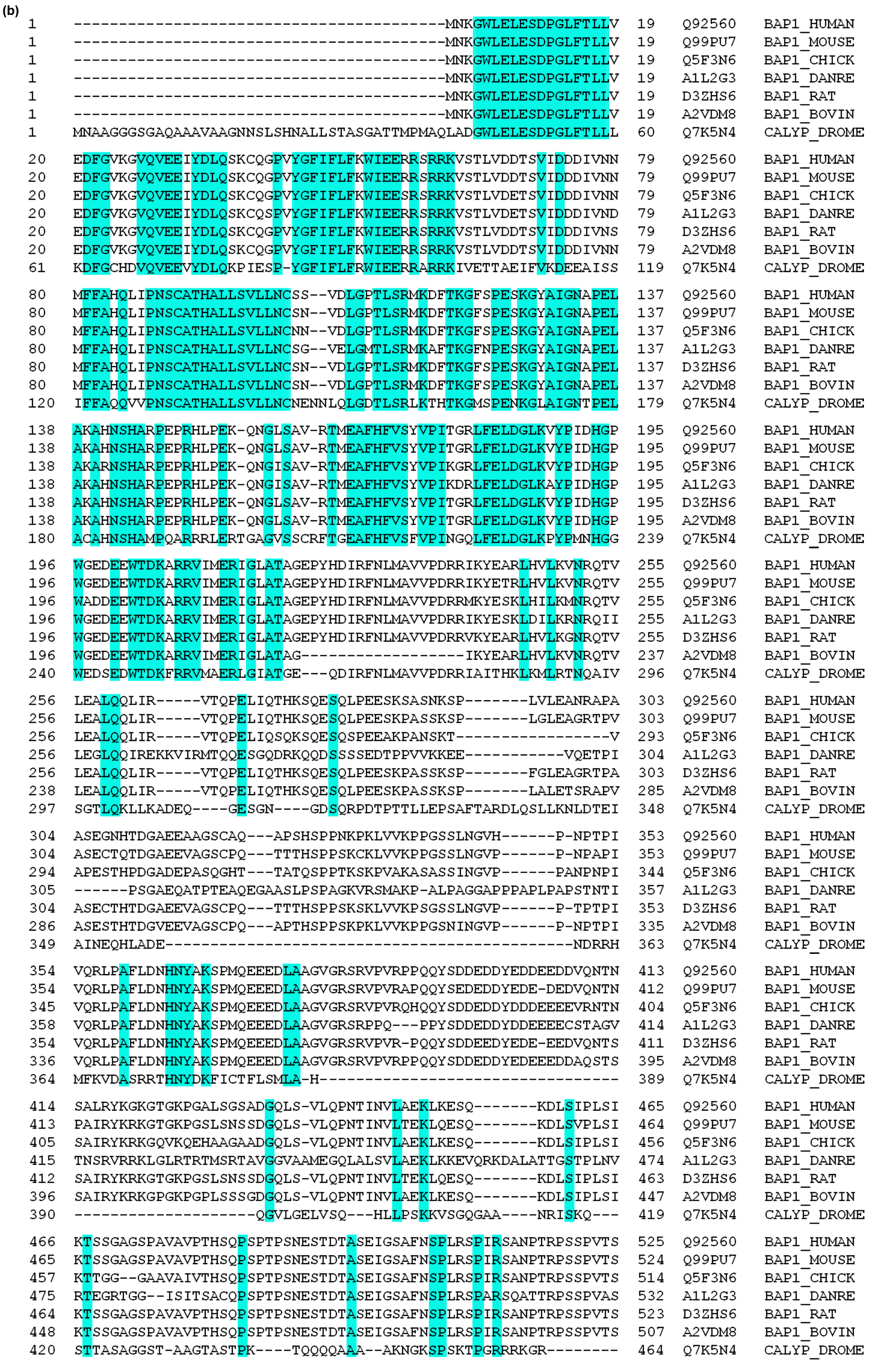


**Supplementary Figure S2**


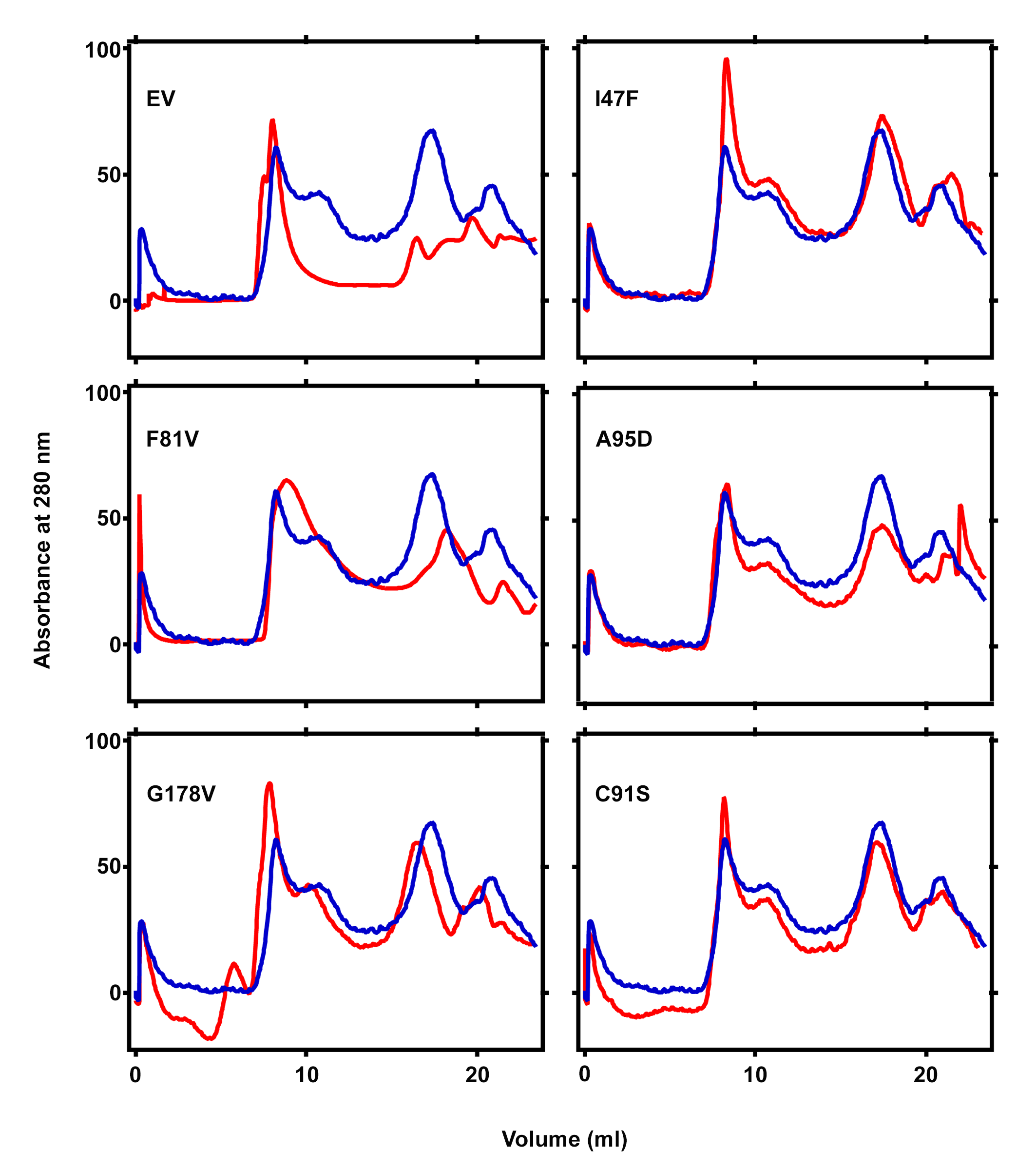


**Supplementary Figure S3**


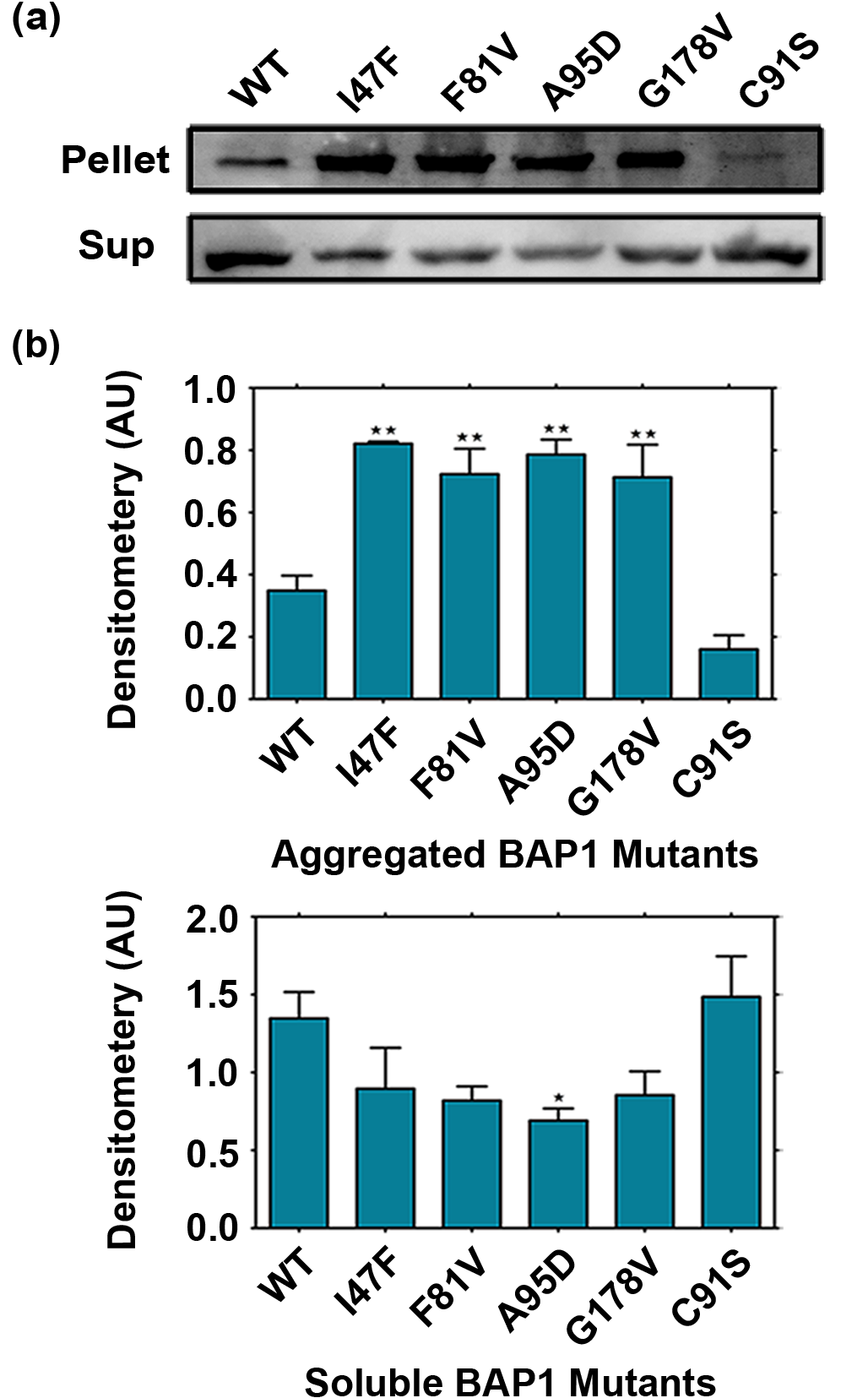


**Supplementary Figure S4**


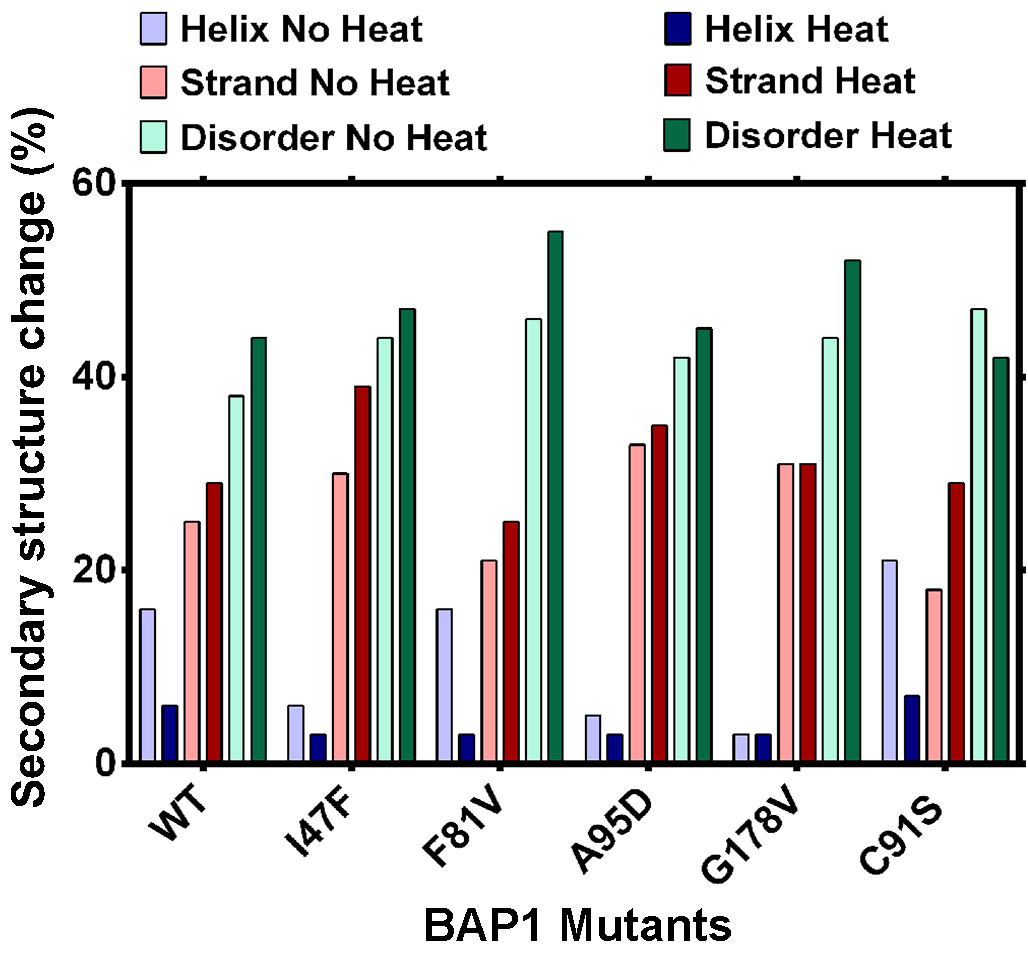


**Supplementary Table 1: Melting point analysis of wild type and mutant BAP1 proteins.**

| **S. No.** | **Name of Protein** | **Melting Point** |
| --- | --- | --- |
|  |  |  |
| 1 | WT 240 | 48.044 |
| 2 | WT FL | 44.018 |
| 3 | I47F | 39.118 |
| 4 | F81V | 46.851 |
| 5 | A95D | 55.875 |
| 6 | G178V | 56.387 |
| 7 | C91S | 49.447 |

**Supplementary Table 2: Primers for qPCR**

| **Gene** | **FORWARD PRIMER** | **REVERSE PRIMER** |
| --- | --- | --- |
| ***TP53I3*** | 5′- CCAGGAGGCCCATAAGTACA -3′ | 5′- CTTCTCCAGGTTTGCTCTGG -3′ |
| ***MCM3*** | 5′- CGCAGTGTCCACTACTGTCC -3′ | 5′- CTCCTGGATGGTGATGGTCT -3′ |
| ***CDKN1B*** | 5′- CATTTGGTGGACCCAAAGAC -3′ | 5′- CTTCTGAGGCCAGGCTTCTT -3′ |
| ***Hsp70*** | 5′- ACCAAGCAGACGCAGATCTTC -3′ | 5′- CGCCCTCGTACACCTGGAT -3′ |
| ***Hsp90*** | 5′-ACTACACATCTGCCTCTGGTGAT -3′ | 5′-TGTTTTCCGAAGACGTTCCACAA -3′ |
| ***GAPDH*** | 5′- GTCTTCACCACCATGGAGAAGGC -3′ | 5’-CATGCCAGTGAGCTTCCCGTTCA -3′ |
